# Intravenous superoxide dismutase as a protective agent to prevent impairment of lung function induced by high tidal volume ventilation

**DOI:** 10.1186/s12890-017-0448-9

**Published:** 2017-07-26

**Authors:** Nan-Chun Wu, Fan-Ting Liao, Hao-min Cheng, Shih-Hsien Sung, Yu-Chun Yang, Jiun-Jr Wang

**Affiliations:** 1 Division of Cardiovascular Surgery, Department of Surgery, Chi-Mei Foundation Hospital, 901, Chung Hwa Rd. Yung Kang, Tainan, Taiwan; 2 School of Medicine, Fu Jen Catholic University, No. 510, Zhongzheng Rd., Xinzhuang Dist, New Taipei City, 24205 Taiwan; 3 Division of Cardiology, Department of Internal Medicine, Taipei Veterans General Hospital, Taipei, Taiwan; 4 Department of Medical Education, Taipei Veterans General Hospital, Taipei, Taiwan; 5 Institute of Public Health and Community Medicine Research Center, National Yang-Ming University, Taipei, Taiwan; 6 Department of Medicine, National Yang-Ming University, Taipei, Taiwan

**Keywords:** Rats, Lung function, Superoxide dismutase, Inflammation, Oxidative stress, Nitric oxide, Positive pressure mechanical ventilation, Surfactant protein A, Surfactant protein D

## Abstract

**Background:**

Positive-pressure mechanical ventilation is essential in assisting patients with respiratory failure in the intensive care unit and facilitating oxygenation in the operating room. However, it was also recognized as a primary factor leading to hospital-acquired pulmonary dysfunction, in which pulmonary oxidative stress and lung inflammation had been known to play important roles. Cu/Zn superoxide dismutase (SOD) is an important antioxidant, and possesses anti-inflammatory capacity. In this study, we aimed to study the efficacy of Cu/Zn SOD, administered intravenously during high tidal volume (HTV) ventilation, to prevent impairment of lung function.

**Methods:**

Thirty-eight male Sprague-Dawley rats were divided into 3 groups: 5 h ventilation with (A) low tidal volume (LTV; 8 mL/kg; *n* = 10), (B) high tidal volume (HTV; 18 mL/kg; *n* = 14), or (C) HTV and intravenous treatment of Cu/Zn SOD at a dose of 1000 U/kg/h (HTV + SOD; *n* = 14). Lung function was evaluated both at baseline and after 5-h ventilation. Lung injury was assessed by histological examination, lung water and protein contents in the bronchoalveolar lavage fluid (BALF). Pulmonary oxidative stress was examined by concentrations of methylguanidine (MG) and malondialdehyde (MDA) in BALF, and antioxidative activity by protein expression of glutathione peroxidase-1 (GPx-1) in the lung. Severity of lung inflammation was evaluated by white blood cell and differential count in BALF, and protein expression of inducible nitric oxide synthase (iNOS), intercellular adhesion molecule-1 (ICAM-1), tumor necrosis factor-α (TNF-α), matrix metalloproteinase-9 (MMP-9), and mRNA expression of nuclear factor-κB (NF-κB) in the lung. We also examined protein expression of surfactant protein (SP)-A and D and we measured hourly changes in serum nitric oxide (NO) level.

**Results:**

Five hours of LTV ventilation did not induce a major change in lung function, whereas 5 h of HTV ventilation induced apparent combined restrictive and obstructive lung disorder, together with increased pulmonary oxidative stress, decreased anti-oxidative activity and increased lung inflammation (*P* < 0.05). HTV ventilation also decreased SP-A and SP-D expression and suppressed serum NO level during the time course of ventilation. Cu/Zn SOD administered intravenously during HTV ventilation effectively reversed associated pulmonary oxidative stress and lung inflammation (*P* < 0.05); moreover, it preserved SP-A and SP-D expressions in the lung and increased serum nitric oxide (NO) level, enhancing vascular NO bioavailability.

**Conclusions:**

HTV ventilation can induce combined restrictive and obstructive lung disorders. Intravenous administration of Cu/Zn SOD during HTV ventilation can prevent lung function impairment and lung injury via reducing pulmonary oxidative stress and lung inflammation, preserving pulmonary surfactant expression, and enhancing vascular NO bioavailability.

## Background

For more than half a century, positive-pressure mechanical ventilation has been regarded as an essential intervention assisting patients with respiratory failure in the intensive care unit and facilitating oxygenation in the operating room. On the other hand, evidence from patient’s studies indicates that mechanical ventilation is one of the primary factors leading to hospital-acquired lung injury, and patients with burn injury, trauma or pre-existing respiratory diseases are particularly at high risks [40]. A recent survey of patients having cardiac surgery revealed that though most patients do not have signs of pulmonary dysfunction or lung injury prior to the surgery, some eventually develop perioperative lung injury [18]. Though the exact mechanism responsible is uncertain, higher tidal volume employed commonly to reduce risks of hypoxemia and pulmonary atelectasis is thought to play a critical role [38].

Pulmonary oxidative stress and lung inflammation had been implicated in the pathogenesis of ventilator-associated pulmonary dysfunction and lung injury [10, 15, 60, 68]. Overinflating alveoli and repeated stretching of lung tissues can produce reactive oxygen species (ROS), and diminish prostanoid synthesis that promotes redox imbalance and cyclooxygenase induction and inflammatory responses [68]. Also, formation of ROS disrupts the regulation of nitric oxide synthase (NOS) causing nitrotyrosine accumulation, irregular tracheal mucus secretion, and increased airway resistance [63]. In addition, stretching lung tissues directly alters metabolism and secretion of pulmonary surfactant proteins (SPs) that mediate alveolar surface tension and lung compliance, while surfactant protein-A (SP-A) and D (SP-D) augment pulmonary immune defense mechanisms and inhibit endogenous lipid peroxidation [4]. Moreover, excessive lung stretch activates nuclear factor-κB (NF-κB) that mediates the production and release of proinflammatory cytokines and chemokines that, in turn, promote adhesion molecule expression [28] and facilitate inflammatory cell infiltration in lung [34]. For over two decades, low tidal volume ventilation has been proposed as a protective strategy; still ventilator-associated lung injury is not uncommon and, thus far, there is still no consensus regarding the optimal ventilation strategy [48]. Terragni et al. [76] reported that even a well-accepted protective ventilation strategy, namely a tidal volume of 6 mL/kg and a plateau pressure ≤ 30 cm H_2_O, may not protect all patients against ventilator-induced pulmonary dysfunction. Consequently, an effective pharmacological intervention may be necessary to protect ventilator-associated pulmonary dysfunction and lung injury.

Superoxide dismutase (SOD) is an important antioxidant, active in endothelial cells, cytoplasm and mitochondrial intermembrane matrix [57]. It protects cells against superoxide damage through catalyzing the dismutation of superoxide radicals into molecular oxygen and hydrogen peroxide [50, 57] and thus inhibits peroxynitrite-mediated oxidative protein modification and cell membrane lipid peroxidation [27]. Moreover, SOD can facilitate vascular function, increasing nitric oxide (NO) bioavailability through competing with NO for superoxide anions [31]. SOD was also shown to inhibit neutrophil-mediated inflammation through regulating neutrophil apoptosis [85]. Despite all the benefits and no major side effects, therapeutic efficacy of SOD treatment on tissue damage, in general, has been limited by its short circulatory half-life and low transcapillary permeability, due to its relatively large molecular size (molecular weight ~ 32 kDa) [84]. On the other hand, intravenous administration of Cu/Zn SOD has been demonstrated to be effective against hyperoxia-induced lung injury [53]. Though the authors did not provide explanation of why SOD treatment was so effective in hyperoxic lung injury, its protective effectiveness is likely related to its substantial permeability across the pulmonary capillary. Increased albumin concentration in the bronchoalveolar lavage fluid (BALF) is the hallmark of hyperoxic lung injury [53] and the molecular weight (∼69 kDa) of albumin is more than twice that of SOD. Albumin leak is also characteristic of ventilator-induced lung injury [80] and we suggest that the observed protective efficacy of intravenously administered SOD may imply substantial transcapillary permeability.

The aim of our study was to determine the protective efficacy of intravenously administered SOD against mechanical ventilation-induced impairment of lung function. Using an in vivo rat model of mechanical ventilation, we showed that 5 h of high tidal volume (HTV) ventilation can induce apparent combined restrictive and obstructive lung disorder and lung injury, evident by increased lung water content and albumin content in BALF. We have identified pulmonary oxidative stress and lung inflammation and reduced expression of pulmonary surfactant protein (SP)-A and D as the main contributors to the impairment of lung function and observed that intravenous administration of Cu/Zn SOD effectively reversed mechanical ventilation-associated detrimental effects. In addition, intravenous administration of Cu/Zn SOD steadily increased the level of serum nitric oxide (NO) during the course of ventilation, suggesting that increased NO bioavailability in pulmonary circulation contributes to the preservation of lung function.

## Methods

### Study protocol

This study was performed on male Sprague-Dawley rats (BioLASCO Co., Taipei, Taiwan) weighing between 250 and 300 g. Rats were housed in a pathogen-free animal facility and food and water were available ad libitum. The study protocol was approved by the Animal Care and Use Committee of the Fu Jen Catholic University and complied with the Guidelines for the Care and Use of Laboratory Animals (NIH Guide, volume 25, Number 28, 1996).

Rats were anesthetized with Zoletil 50 (50 mg/kg; i.p., Virbac, Carros cedex, France). To reduce mucous secretion, animals were given atropine (0.8 mg/kg; i.m.; Sigma, MO, USA) 15 min prior to the anesthesia. The depth of anesthesia was monitored every 30 min by assessing the reflex withdrawal response to pinching of the hind paws. A tracheotomy was performed and the trachea was cannulated with a sterile polystyrene catheter (PE240), which in turn was connected to a small animal ventilator (TOPO Small Animal Ventilator, Kent Scientific, CT, USA; constant-frequency, volume-controlled). Airway pressure was monitored continuously (Deltran 6069, Utah Medical Inc., Utah, USA). The right jugular vein was cannulated with a polystyrene catheter (PE50) through which Cu/Zn SOD or equal amounts of normal saline (0.9%) were administered. Body temperature was maintained at ~37 °C using a heating blanket. The left femoral artery was cannulated by a polyethylene catheter (PE50), through which aortic pressure (P_Ao_) was monitored (Deltran 6069). Blood samples were collected every 60 min for assessments of blood gases (Radiometer ALB 5, Brønshøj, Denmark), blood count (Fujifilm Sericol Retarder ZV558, Osaka, Japan), and serum NO level. Hemodynamic data were recorded at 2000 Hz using a 16-channel data acquisition system (model MP150, Biopac Systems Inc., CA, US) and stored using a dual-processor laptop computer.

Thirty-eight rats were ventilated for 5 h, either with low tidal volume (LTV) of 8 mL/kg (*n* = 10) or high tidal volume (HTV) of 18 mL/kg. The inspiratory/expiratory ratio for those ventilated with LTV and HTV were 0.25 and 0.45, respectively. Other ventilatory parameters including positive end expiratory pressure (PEEP = 0 cmH_2_O; positive PEEP has been shown to have lung protective effectiveness [1]) fraction of inspired oxygen (0.21), respiratory rate (60 stroke/min) were identical between groups. HTV-ventilated rats were administered intravenously either with Cu/Zn SOD (HTV + SOD, *n* = 14) or with an equal amount of saline (HTV; *n* = 14); the same amount of saline was also administered intravenously to LTV-ventilated rats. Human erythrocyte Cu/Zn SOD (S9636-15KU, Sigma-Aldrich Co., MO, US) was administered at a dose of 1000 U/kg/h via a high-precision syringe pump (KD Scientific, Holliston, MA, USA) during the entire course of ventilation. The dose adopted was determined in a preliminary study, in which 4 doses (200, 500, 1000 and 2000 U/kg/h) were administered during HTV ventilation, the outcome being assessed by means of lung function testing, and concentrations of methylguanidine (MG) and malondialdehyde (MDA) in BALF. Results were dose-dependent, but no major differences were observed between rats treated with 1000 U/kg/h and those with 2000 U/kg/h. In order to maintain the blood pH within 7.30–7.45 during HTV ventilation, an external dead space was added by increasing the volume of intubation tubing [61]. In another study, heat denatured Cu/Zn SOD (1000 U/kg/h) was treated during HTV ventilation; the results of lung function testing and tissue MDA level were compared to those assessed in the HTV group and showed no significant difference, suggesting no protective effectiveness of heat denatured Cu/Zn SOD. At the end of study, rats were euthanized by an overdose of anesthetic (200 mg/kg i.p). The lungs and trachea were removed for BALF measurements, assessments of lung water and histological analysis, and subsequent analyses of protein and mRNA expression.

### Lung function testing

Lung function testing was conducted both at baseline and after 5 h of mechanical ventilation using a Buxco forced-maneuver system (Buxco Research Systems, Wilmington, NC, USA), comprised of a plethysmograph chamber, a control panel and pressure and vacuum reservoirs. Anaesthetized rats were placed inside the plethysmograph chamber with their tracheal cannulae connected to the breathing valve. The plethysmograph was connected to a differential pressure transducer and an amplifier system. After adjusting the respiratory rate of the system ventilator to match that of the animal, a series of forced maneuvers mimicking spirometric maneuvers in human subjects (i.e., inflating, occluding, and deflating the lungs at different rates) were conducted and parameters of lung function (i.e., lung volumes, airway flows and lung resistance and compliance) were calculated via the Biosystem XA system (Buxco) and exported to text files. Pulmonary function testing at each status (baseline or post-mechanical ventilation) was repeated three times to yield averaged measurements.

### Lung tissue preparation

Lungs and trachea removed at the end of experiment were weighed immediately. BALF was acquired from the left lung; the right lung was used for histological examination (upper lobe), measurement of lung water (middle lobe), and analysis protein expression or mRNA (lower lobe). The whole lung was inflated to 20 cmH_2_O and the right main bronchus was ligated to separate the left from the right lung. The right upper lobe bronchus was then ligated and the inflated upper right lobe was excised and immersed in 10% buffered formalin at 4 °C for 24 h for tissue fixation. The middle right lobe was weighed upon dissection (wet weight) and weighed again after being dehydrated in an oven at 70 °C for 7 days (dry weight). The lower lobe of right lung was sectioned and frozen immediately in liquid nitrogen and stored in a -80 °C freezer for subsequent analysis of protein and mRNA expression.

The upper lobe of right lung was processed following dehydration (TP1020, Leica Biosystems, Richmond, IL, USA), clearing, paraffin infiltration and embedding. Lung specimens were sectioned (~5 μm-thick; Jung. RM2045, Leica Biosystems), stained with hematoxylin and eosin, and examined under a light microscope. Photo images were taken by a C-mount microscope camera (Whited Inc., Taipei, Taiwan).

### Bronchoalveolar lavage fluid acquisition

BALF was acquired by gently flushing the left lung three times through the trachea with a 1.25 mL aliquot of 0.9% saline pre-warmed to 37°C. A volume of 0.92±0.10 mL of lavage fluid was recovered. Lavage samples were centrifuged at 800 g at 4°C for 10 min. The cell pellets were used for differential cell count and the supernatant was extracted for biomarker analyses.

### Measurement of protein concentration in the BALF

Protein concentration (μg/mL) was measured in the supernatant of BALF using spectrophotometry (Multiskan FC, Thermo Scientific, CA, USA) with an emission wavelength at 630 nm, calibrated by a standard curve for albumin (Sigma).

### Methylguanidine in the bronchoalveolar lavage fluid

The level of MG formation in the BALF has been used as an index for pulmonary hydroxyl radical formation [74]. The lavage supernatant was diluted 1:100, and MG was measured using a spectrofluorimetric detector (Jasco 821-FP Fluorescence Detector, Tampa, FL, USA) with a fluorescent excitation maximum at 395 nm and emission maximum at 500 nm. The assay was calibrated by a standard curve of authentic MG (Sigma M0377, St. Louis, MO, USA), generated by various concentrations of MG (0, 50, 100, 150, and 200 mg/mL). The coefficient of variance in percentage was 3.8% with a detection limit of 1 mg/mL.

### Malondialdehyde levels in the bronchoalveolar lavage fluid

The MDA concentration in BALF has been used as an index for pulmonary oxidative stress and lipid peroxidation [66]. MDA was measured in the supernatant of BALF using enzyme-linked immunosorbent assay (ELISA) kits (ab46070; Abcam, Cambridge, MA, USA) with a detection range of 0.3–65 nmol/mL and a sensitivity of 0.208 nmol/mL. Each sample was performed in duplicate and determined by an automated ELISA reader at 450/540 nm wavelength.

### Total white blood cell count and differential in BALF

Total white blood cell (WBC) count was performed by loading 10 μL of BALF onto a hemocytometer, and examined under light microscopy (400X, BX-40, Olympus, Tokyo, Japan). Differential cell count was conducted by loading 10 μL of the cell pellets on a slide, stained with Liu’s stain, and examined under light microscopy (1000X, Olympus). One hundred cells were examined in each specimen.

### Measurement of plasma nitric oxide level

The level of plasma NO was evaluated by the concentrations of nitrate and nitrite (metabolites of nitric oxide) using a high-performance liquid chromatography system (ENO-20, Eicom Nox Analyzer, Kyoto, Japan), which has a sensitivity of 30 pmol for nitrate and nitrite anion. Blood samples mixed with an equal amount of methanol were centrifuged at 15,000 g at 4°C for 10 min. The top supernatant, filtered through an ultrafine membrane with a cut-off protein molecular weight of 3 kDa, was injected into the HPLC system. Samples were separated through a strong anion-exchange column (Spherisorb SAX, 250 × 4.6 mm with internal diameter of 5 μm) followed by a nitrate-to-nitrite reduction process and a Griess diazotization reaction. The chromophore was detected at the wavelength of 540 nm with the coefficient of variance of 3.2% and detection limit of 2 pmol.

### Western blot analysis

Frozen lung tissues of around 200 mg were homogenized in a lysis buffer (10 mM Tris · HCl, pH 7.5, 1% Triton X-100, 1 mM EDTA, 1 mM PMSF, 10 μg/mL aprotonin, and 10 μg/mL leupeptin). The total protein concentration was determined using the Bicinchoninic Acid protein assay kit (Sigma-Aldrich, St. Louis, MO, USA), measured at the absorbance wavelength of 562 nm and calibrated to a standard curve. The sample’s molecular weight was determined using the Precision Plus Protein Kaleidoscope standards (10 μl, Bio-Rad). The protein extraction, mixed with an equal amount of sodium dodecyl sulphate (SDS) buffer, was heated to 95 °C for 5 min and separated on a 10% SDS polyacrylamide gels in a running buffer at 80 V. These spatially separated proteins were transferred onto a nitrocellulose membrane (Bio-Rad) at 100 mA and at 4°C overnight. Non-specific binding was blocked through placing the membrane in a blocking buffer (HyCell Biotechnology Inc. Raleigh, NC, USA) at room temperature for 1 min. Blots were then incubated with the primary antibody at 4 °C on an orbital shaker. In this study, the primary antibodies were glutathione peroxidase-1 (GPx-1; ab22604, Abcam, Cambridge, UK), tumor necrosis factor-α (TNF-α; ab1793, Abcam), iNOS (ab15323, Abcam), intercellular adhesion molecule-1 (VCAM-1; MR106, Novus Biologicals, Littletown, CO, USA), matrix metalloproteinase-9 (MMP-9; ab7299, Abcam), and surfactant protein A (SP-A; ab115791, Abcam) and D (SP-D; ab15687, Abcam). Antibodies were diluted according to the instructions before use. The membranes were washed for 10 min in Tris-Buffered Saline Tween-20 (TBST) three times, followed by incubation in a diluted horseradish peroxidase-conjugated secondary antibody (EMD Millipore Corp, Billerica, MA, USA) at room temperature for 1 h. After incubation, the membrane was washed 10 min three times using TBST. Blots were developed with the detection reagents (EMD Millipore, Darmstadt, Germany), and quantified using a gel documentation and image-analysis software (MiniChemi, SageCreation, Beijing, China).

### Real-time PCR analyses

The mRNA of lung tissue was isolated using the RNAzol reagent (Molecular Research Center, OH, USA), followed by treatment of RNase-free DNase (Qiagen kit) during column purification. Reverse transcription of total mRNA to cDNA was carried out using the First Strand cDNA synthesis kit (avian myeloblastosis virus; Roche Applied Science, Penzberg, Bavaria, Germany) and random primers. This cDNA was then used as a template for the gene-specific primer of real-time PCR. Real-time PCR was conducted using Platinum SYBR Green qPCR Supermix-UDG (Invitrogen Carlsbad, NM, USA) and an ABI PRISM 7500 real time PCR system (Applied Biosystems, Drive Foster City, CA, USA). Forward and backward primer sequences for NF-κB were CCGGGCAGGTCTCAGC and GGGCTGCTCAATGATCTCCA, respectively, designed using Primer Express V.2.0 (Applied Biosystems) software. The thermal cycling conditions for real-time PCR were set at 50°C for 2 min, followed by 95°C for 10 min, and 40 cycles of melting at 95°C for 15 s, and annealing and extension at 60°C for 60 s. Levels of mRNA relative to an endogenous control (β-actin) were calculated by the ΔΔC_T_ method, using 7500 System SDS software version 1.2.1.22 (Applied Biosystems).

### Statistical analysis

Data were presented as mean±SEM. Comparisons across two sets of groups were analyzed with two-way analysis of variance, followed by Tukey’s post-hoc test. *P* < 0.05 is considered statistically significant.

## Results

The hourly recorded mean aortic pressure (P_mean_), peak airway pressure (P_paw_) and blood gas data (pH, PCO_2_ and PO_2_) are summarized in Table 1. Five hours of LTV ventilation did not induce significant changes in P_mean_, P_paw_ or blood gas data versus those of the baseline. Blood gas at baseline was not statistically different between groups. Without addition of dead space, HTV ventilation induces apparent respiratory alkalosis and hyperventilation. Dead space addition (~3.0 mL) helped sustain blood pH, mostly within a range of 7.30 to 7.45, during HTV ventilation, as well as improving respiratory alkalosis and hyperventilation. Decreased PaO_2_ and increased PaCO_2_ during the last hour of HTV ventilation indicates impaired gas exchange, which is consistent with the interstitial edema and hyaline membrane formation observed in histological examination (see below). On the other hand, SOD treatment was demonstrated to reverse the impairment of gas exchange. HTV ventilation decreased P_mean_ by 6–10 mmHg as compared with the baseline, and P_mean_ remained relatively stable during the course of ventilation, with or without SOD treatment.

**Table 1:** Mean arterial pressure (P_mean_), peak airway pressure (P_paw_) and blood gas measured during the course of 5h of mechanical ventilation

		P_mean_ (mmHg)	P_paw_ (mmHg)	PaO_2_ (mmHg)	PaCO_2_ (mmHg)	pH
LTV	Baseline	122.8*±5.7*	12.0*±0.2*	104.6*±4.8*	38.1*±5.2*	7.36*±0.2*
	1 h	121.2*±6.4*	12.1*±0.2* ^*^	114.7*±7.6*	42.3*±6.3*	7.34*±0.3*
	2 h	118.7*±6.9*	12.0*±0.1* ^*^	110.3*±6.6*	33.3*±5.5*	7.39*±0.2*
	3 h	124.3*±7.1*	11.9*±0.2* ^*^	112.8*±8.6*	35.3*±6.2*	7.43*±0.3*
	4 h	114.8*±8.5*	12.0*±0.1* ^*^	108.8*±7.1*	36.3*±4.8*	7.41*±0.2*
	5 h	123.6*±7.5*	12.0*±0.2* ^*^	113.2*±6.7*	43.3*±4.4*	7.38*±0.2*
HTV	Baseline	121.3*±7.7*	12.2*±0.2*	*102.2±6.1*	43.1*±3.5*	7.32*±0.4*
	1 h	116.6*±7.8*	21.9*±0.2* ^*^	118.5*±7.4*	48.8*±4.1*	7.38*±0.3*
	2 h	114.7*±5.4*	22.0*±0.2* ^*^	120.8*±7.7*	53.1*±3.7*	7.41*±0.4*
	3 h	117.3*±6.8*	21.9*±0.2* ^*^	114.9*±10.0*	47.8*±4.3*	7.34*±0.5*
	4 h	112.4*±6.2*	22.2*±0.2* ^*^	101.1*±10.1*	56.8*±3.6*	7.36*±0.4*
	5 h	108.4*±5.7*	22.0*±0.2* ^*^	84.2*±10.4* ^*^	66.1*±5.0* ^*^	7.26*±0.5*
HTV + SOD	Baseline	*124.8±7.4*	12.2*±0.2*	97.7*±6.4*	41.6*±3.4*	7.40*±0.4*
	1 h	114.2*±5.2*	22.0*±0.2* ^*^	122.5*±8.2*	43.1*±6.6*	7.43*±0.3*
	2 h	115.7*±4.5*	22.1*±0.2* ^*^	115.1*±7.8*	50.1*±4.4*	7.39*±0.5*
	3 h	110.3*±8.4*	21.5*±0.2* ^*^	122.1*±6.8*	47.1*±5.6*	7.34*±0.4*
	4 h	113.0*±7.3*	22.2*±0.2* ^*^	107.1*±6.8*	55.1*±4.5*	7.34*±0.5*
	5 h	116.1*±7.7*	22.1*±0.2* ^*^	98.1*±6.8*	53.7*±5.4*	7.32*±0.5*

### Cu/Zn SOD protects against HTV ventilation induced damages of lung structure

**Fig. 1:**
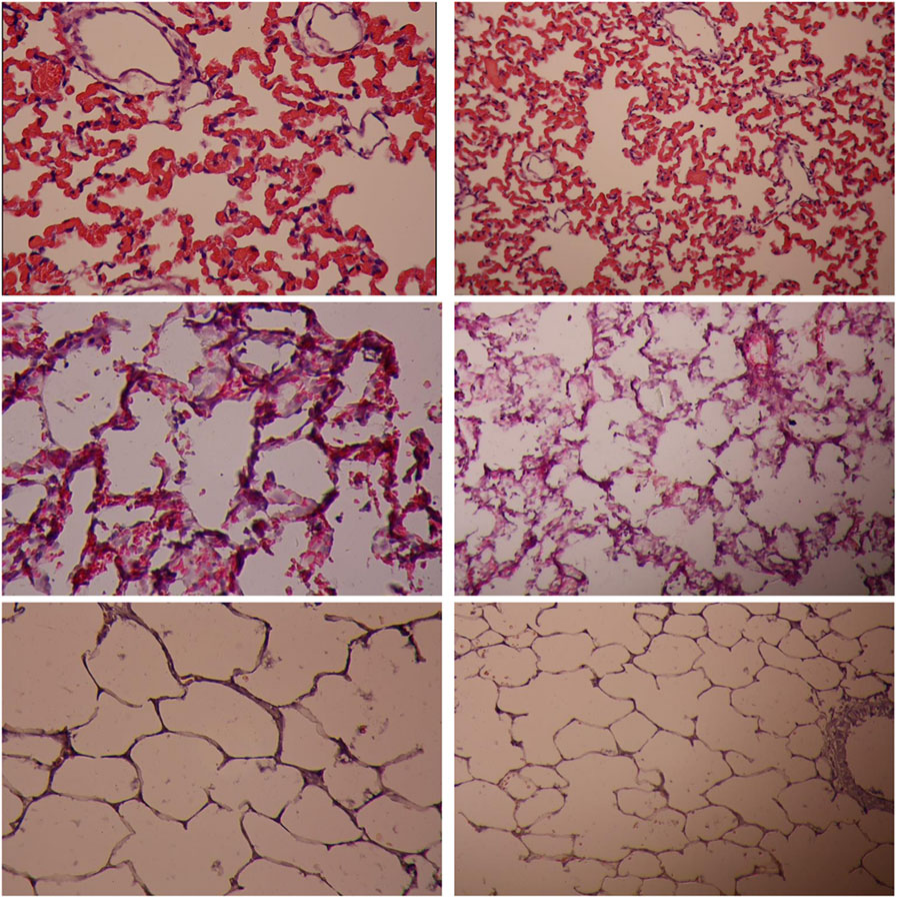
Representative images of HE-stained lung tissues. Images magnified by 400X are on the *left panel* and magnified by 100X shown on the *right; from top to bottom panels*, LTV, HTV and HTV + SOD group

Figure 1 shows qualitative comparison of representative images of HE-stained lung tissues, magnified by 400X (left) and 100X (right); from top to bottom, the LTV, HTV and HTV + SOD group. LTV-ventilated lungs exhibit clear open alveolar spaces, and well-defined pneumocytes with no apparent infiltration of inflammatory cells. In contrast, HTV-ventilated lungs feature diffuse alveolar hemorrhage, interstitial edema, hyaline membrane formation and inflammatory cells infiltration in the pulmonary parenchyma. Treatment with Cu/Zn SOD during HTV ventilation ameliorated ventilator-induced alveolar hemorrhage, interstitial edema and hyaline membrane formation, and also decreased the level of inflammatory cell infiltration.

### Cu/Zn SOD protects against HTV ventilation-induced lung function impairment

**Fig. 2:**
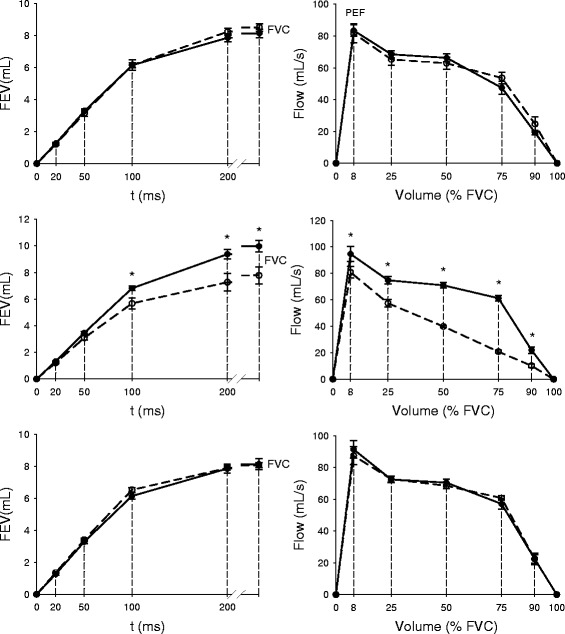
Results of lung function testing of the three study groups, by means of forced expiratory volume (FEV) as a function of time (*left panel*) and expiratory flow volume curve (*right panel*); *from top to bottom panel*, LTV, HTV and HTV + SOD group (data at baseline are presented by *closed symbols* and *solid line*, and data at post-ventilation were presented by *open symbols* and *dashed line*) (* and # signify *P* < 0.05 and *P* < 0.001, respectively)

Figure 2 shows results of lung function testing. Forced expiratory volume (FEV) as a function of time is shown in the left panel and expiratory flow volume in the right panel; from top to bottom, the LTV, HTV and HTV + SOD group. Five hours of LTV ventilation did not significantly alter FEV or expiratory flow volume relationship versus those assessed at baseline, whereas 5 h of HTV ventilation markedly changed lung function, evident by decreased FEV at 100 (FEV_100_), 200 (FEV_200_) milliseconds and forced vital capacity (FVC), and decreased forced expiratory flow at 25%, 50%, 75% and 90% of FVC (FEF_25_, FEF_50_, FEF_75_ and FEF_90_) and peak expiratory flow (PEF) versus the baseline (*P* < 0.05).

**Fig. 3:**
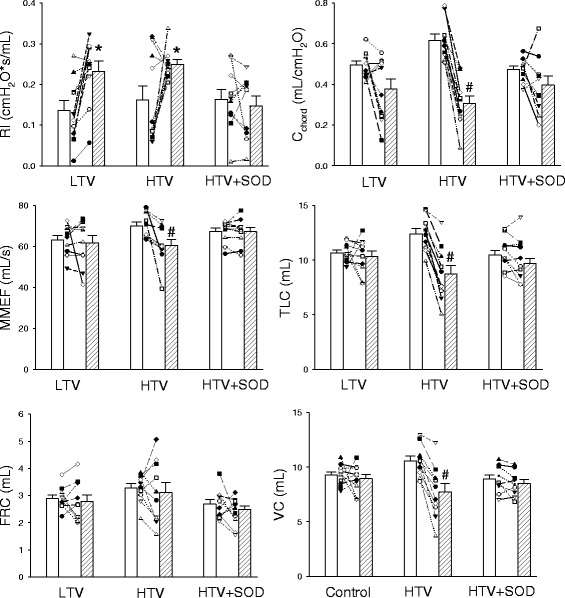
Lung mechanics assessed at baseline and after 5 h ventilation. Post-ventilation is presented by *hatched bar* and baseline by *open bar*; direct measurements are represented by *connected symbols*. Five hours of LTV ventilation altered inspiratory resistance (RI), but did not significantly change chord compliance (C_chord_), maximum mid-expiratory flow (MMEF), total lung capacity (TLC), vital capacity (VC) or functional residual capacity (FRC) versus the baseline. Five hours of HTV ventilation alter all parameters of lung function. Cu/Zn SOD treatment minimized deterioration of lung function. (* and # signify *P* < 0.05 and *P* < 0.001, respectively)

Figure 3 shows lung mechanics assessed at baseline (open bar) and after 5 h ventilation (hatched bar); direct measurements are represented by connected symbols. Five hours of LTV ventilation increased inspiratory resistance (RI) (*P* < 0.05), but did not alter other parameters of lung function versus the baseline. In contrast, 5 h of HTV ventilation markedly increased RI (*P* < 0.05), while decreased maximum mid-expiratory flow (MMEF)(*P* < 0.001), chord compliance (C_chord_)(*P* < 0.001), vital capacity (VC) (*P* < 0.001) and total lung capacity (TLC)(*P* < 0.001) versus the baseline. Collectively, HTV ventilation induces combined restrictive and obstructive lung disorder.

In the bottom panel of Fig. 2 and in Fig. 3, we showed that intravenous treatment of Cu/Zn SOD during HTV ventilation prevented impairment of lung function and lung mechanics, evident by sustained FEV, expiratory flow volume relationship, RI, MMEF, C_chord_, TLC and VC relative to the baseline.

### Cu/Zn SOD reduces HTV ventilation-induced increases in trans-capillary protein permeability and lung water content

**Fig. 4:**
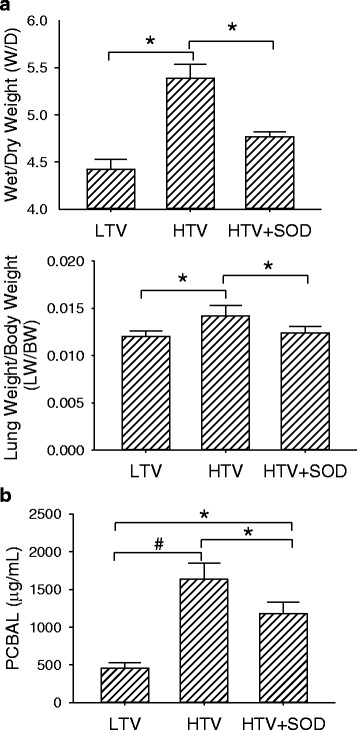
**a** HTV ventilation increases the lung wet-to-dry weight ratio (W/D) (*upper panel*), and lung-weight-to-body weight ratio (LW/BW) (*lower panel*) as compared with those assessed in the LTV group. **b** HTV ventilation significantly increased the protein concentration in bronchoalveolar lavage fluid (PCBAL). Treatment with SOD notably decreased HTV ventilation-associated increases in W/D and LW/BW ratio, and decreased PCBAL (* and # signify *P* < 0.05 and *P* < 0.001, respectively)

Figure 4 shows that 5 h of HTV ventilation markedly increased the content of lung water as compared with the LTV group, evident by increased wet-to-dry lung weight ratio (W/D; 5.39 ± 0.15 vs. 4.42 ± 0.11; *P* < 0.05) (upper panel), and lung-weight-to-body-weight ratio (LW/BW; 0.0142 ± 0.0011 vs. 0.0120 ± 0.0006; *P* < 0.05) (lower panel). Moreover, HTV ventilation significantly increased the protein concentration in BALF (PCBAL; 1635±212 vs. 459±69 μg/mL; *P* < 0.001) (Fig. 4b). Intravenous treatment of Cu/Zn SOD notably decreased HTV ventilation-induced increases in W/D (4.77 ± 0.05; *P* < 0.05) and LW/BW ratio (0.0124 ± 0.0007; *P* < 0.05), and reduced PCBAL (1077.5 ± 51.6 μg/mL; *P* < 0.05).

### Lung lavage white blood cell count and differential

**Fig. 5:**
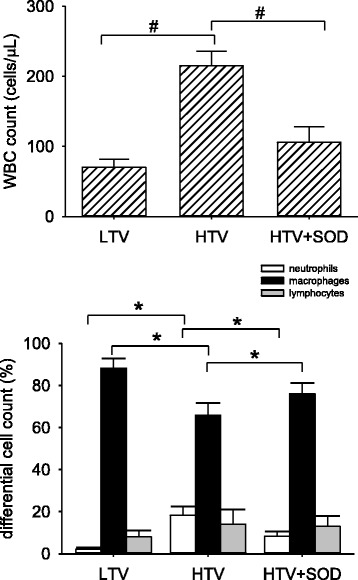
HTV ventilation markedly increased lavage content of WBC versus the LTV group. HTV ventilation increases percent neutrophils in BALF. Though absolute macrophages (*lower panel*) increased after HTV ventilation, the percent macrophages decreased. SOD treatment during HTV ventilation markedly reduced total white blood cells and the percentage of neutrophils in BALF (* and # signify *P* < 0.05 and *P* < 0.001, respectively)

White blood cell (WBC) count and differential in the lung lavage has been widely used as an indicator of lung inflammation [78]. Figure 5 shows that HTV ventilation markedly increased lavage content of WBC versus the LTV group (215±21 vs. 70±14 cells/μL; *P* < 0.05) (upper panel), and increased the percentage of neutrophils (2.2±0.4 vs. 18.4±4.2%; *P* < 0.05) (white bar; lower panel). Though absolute macrophages increased after HTV ventilation, the percentage of macrophages decreased (88.2±4.7 versus 65.8±6.8%; *P* < 0.05) and the percentage of lymphocytes moderately increased (8.3±3.1 versus 14.3±6.9%) (black and grey bar, respectively; lower panel). Intravenous treatment of Cu/Zn SOD during HTV ventilation notably reduced total white blood cells (106±23 cells/μL) and the percentage of neutrophils (8.4±2.2%) in BALF, suggesting decreased lung inflammation.

### High tidal volume ventilation increases concentrations of malondialdehyde and methylguanidine in the bronchoalveolar lavage fluid

**Fig. 6:**
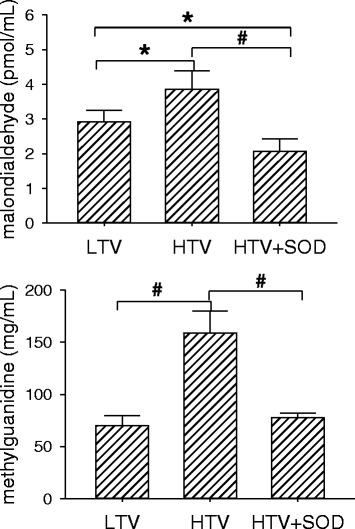
Five hours of HTV ventilation increases MDA and MG concentrations in BALF as compared with the LTV group. Cu/Zn SOD treatment during HTV ventilation effectively reduces MDA and MG in BALF (* and # signify *P* < 0.05 and *P* < 0.001, respectively)

Malondialdehyde (MDA) is an endogenous end-product of oxygen radical-induced and enzymatic lipid peroxidation, and has been used as a biomarker of oxidative stress [29]. The lavage level of methylguanidine (MG), an end-production of protein catabolism, has been associated with the degree of pulmonary hydroxyl radical formation [88]. Figure 6 demonstrates that 5 h of HTV ventilation increased MDA (3.85 ± 0.53 vs. 2.92 ± 0.34 nmol/mL; *P* < 0.05) and MG (158.5 ± 21.1 vs. 70.1 ± 9.6 mg/mL; *P* < 0.001) concentrations in BALF as compared with those of the LTV group. Intravenous SOD treatment during HTV ventilation effectively reduced MDA (2.095 ± 0.35 nmol/mL; *P* < 0.05) and MG (77.5 ± 4.4 mg/mL; *P* < 0.05) in the lung lavage, indicating reduced oxidative stress and ameliorated hydroxyl radical formation and pulmonary lipid peroxidation.

### SOD treatment increases serum level of nitric oxide

**Fig. 7:**
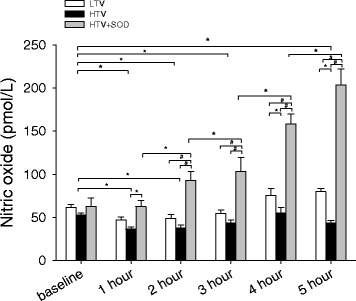
Hourly measured serum NO during LTV (*white*) and HTV ventilation (*black*) and HTV ventilation with SOD administration (*grey*). Both LTV and HTV ventilation decreased serum NO. LTV ventilation decreased serum NO primarily during the first 2 h of ventilation (*P* < 0.05), whereas HTV ventilation decreased serum NO throughout most of the 5 h of ventilation (*P* < 0.05, except at 4 h). Notably, SOD administration monotonically increased serum NO level during the course of HTV ventilation

Nitric oxide (NO) plays an essential role in the modulation of pulmonary vascular, airway smooth muscle tone, non-adrenergic and non-cholinergic neurotransmission and mediation of the inflammatory response [86]. Figure 7 shows hourly measured serum NO in the LTV (white), HTV (black) and HTV + SOD group (grey). We observed that though both LTV and HTV ventilation decreased serum NO, LTV ventilation decreased serum NO primarily during the first 2 h of ventilation (*P* < 0.05), whereas HTV ventilation decreased serum NO throughout most of the 5-h ventilation (*P* < 0.05). Notably, SOD administration monotonically increased serum level of NO during the course of HTV ventilation.

### Western blot analyses of protein expressions of glutathione peroxidase-1 (GPx1), vascular cell adhesion molecule 1 (VCAM-1), inducible nitric oxide (iNOS), matrix metalloproteinase-9 (MMP-9) tumor necrosis factor-α (TNF- α) and surfactant protein A (SP-A) and D (SP-D) in lung

**Fig. 8:**
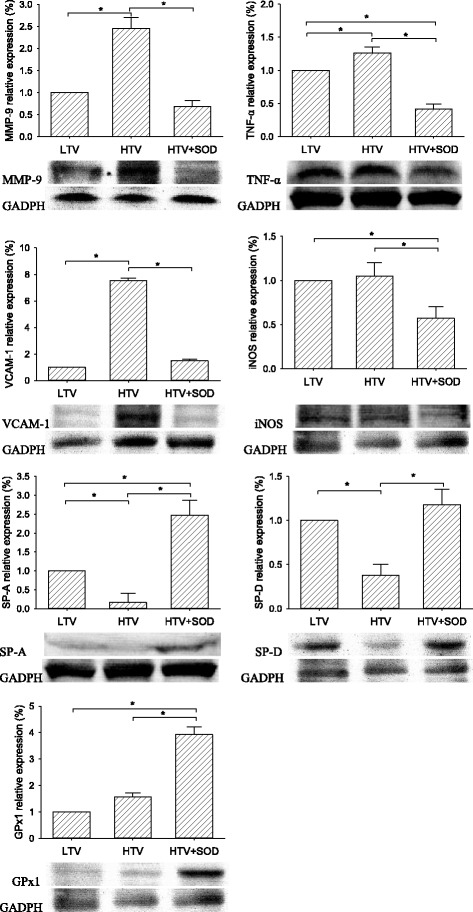
Western blot analyses of protein expressions of glutathione peroxidase-1 (GPx1), vascular cell adhesion molecule 1 (VCAM-1), inducible nitric oxide (iNOS), nuclear factor (NF-κB), matrix metalloproteinase-9 (MMP-9), and surfactant protein A and D (SP-A and SP-D) in lung (*n* = 3; * and # signify *P* < 0.05 and *P* < 0.001, respectively)

We assessed the level of pulmonary antioxidant defense via the protein expression of glutathione peroxidase-1 (GPx1) [70], and the degree of lung inflammation by means of protein expressions of vascular cell adhesion molecule-1 (VCAM-1; it mediates the adhesion of lymphocytes, monocytes, eosinophils and basophils to vascular endothelium), and inducible nitric oxide synthase (iNOS; it functions as a downstream inflammatory mediator of NF-κB [6]), and matrix metalloproteinase-9 (MMP-9; it activates extracellular matrix remodeling and facilitates inflammatory cell recruitment across the epithelium [9]), and surfactant protein A and D (SP-A and SP-D; they mediate pulmonary innate immune function [47] and the latter has been demonstrated to modulate ventilator-induced lung inflammation [67]), and tumor necrosis factor-α (TNF-α; it functions as an essential inflammatory mediator that exerts its effector actions through activation of a transcription factor), and mRNA expression of nuclear factor-κB (NF-κB; it plays a role in inflammation-associated cell death) [83]. Figure 8 shows that HTV ventilation markedly increased VCAM-1, TNF-α and MMP-9 (*P* < 0.05), while decreased SP-A and SP-D expressions in lung (*P* < 0.05) as compared with those assessed in the LTV group (*n* = 3 each). Intravenous SOD administration effectively attenuated HTV ventilation-associated increases in protein expressions of VCAM-1, TNF-α and MMP-9, while improved suppressed protein expressions of SP-A and SP-D in lung (*P* < 0.05). Although lung tissue expressions of GPx-1 and iNOS in the HTV group were not significantly different from those measured in the LTV group, intravenous SOD administration markedly increased the protein expression of GPx-1 while decreased protein expression of iNOS (*P* < 0.05), suggesting improved antioxidant defense and reduced inflammation in lung.

### Messenger RNA expressions of nuclear factor-κB (NF-κB) in lung

**Fig. 9:**
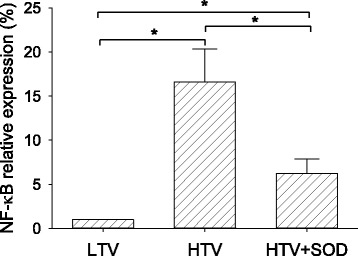
mRNA expression of nuclear factor-κB (NF-κB) (*n* = 3) (*and # signify *P* < 0.05 and *P* < 0.001, respectively)

As a pro-inflammatory transcription factor, nuclear factor-κB (NF-κB) plays an important role in regulating the immune and inflammatory response through upregulating iNOS, MMPs, adhesive molecules, proinflammatory cytokines and chemokines [75]. Figure 9 shows that HTV ventilation upregulated NF-κB mRNA expression by more than 16-fold versus that of the LTV group (*P* < 0.05), whereas intravenous SOD administration effectively reduced HTV ventilation-induced activation of NF-κB, by merely 6-fold that of the LTV group (*P* < 0.05), therefore limiting inflammatory response.

## Discussion

Using this in vivo rat model, we demonstrated that 5 h of LTV ventilation (8 mL/kg) increases RI, but did not significantly alter other parameters of lung function as compared to the baseline, whereas 5 h of HTV ventilation (18 mL/kg) can induce apparent combined restrictive and obstructive lung disorder. In addition, HTV ventilation significantly increased levels of pulmonary hydroxyl radical formation and lipid peroxidation, inflammatory cell infiltration and sequestration, and markedly increased lung inflammation. We showed that intravenous administration of SOD during HTV ventilation minimizes deterioration of lung function and significantly reduces associated lung inflammation and oxidative stress. To our knowledge, this is the first demonstration of apparent protective effectiveness of intravenous SOD in mechanical ventilation, which may have future therapeutic significance.

Cu/Zn SOD is a homodimeric metalloenzyme with two 16.3 kDa 153 amino acid subunits [50]. Superoxide is the predominant free radical produced in the biological system, and SOD exerts enzymatic protection against superoxide damage by degrading superoxide radicals into oxygen and hydrogen peroxide, which in turn are converted by GPx-1 into water [84]. Fukai and Ushio-Fukai [79] reported that SODs inhibit superoxide-induced inactivation of iron-sulfur containing enzymes, such as aconitase and fumarase, and reduce the release of iron and subsequent formation for highly toxic hydroxyl radical or related iron-associated reactive species through reacting with H_2_O_2_. SOD has no apparent side effects and has been shown to possess anti-inflammatory capacity against neutrophilic activity, in addition to being an antioxidant [49]. Reitsma et al. [64] reported that negatively charged vascular endothelial glycocalyx tends to bind extracellular SOD and facilitates its protective role. However, as a therapeutic agent, SOD is limited by its relatively large molecular size and a short circulating half-life of ~6 min [57]. Nevertheless, exogenous treatment of SOD has been demonstrated protective against hyperoxic lung injury. Mikawa et al. [53] showed that intravenous administration of Cu/Zn SOD markedly reduces hyperoxia-induced pulmonary capillary protein and water leak and decreases tissue oxidative stress and inflammation in rabbit lung. In hyperoxic mice, Yen et al. [87] demonstrated that aerosol administration of Cu/Zn SOD improves survival, reduces lung edema and parenchymal damage, and reduces systemic oxidative stress.

Comparable to the protective efficacy of Cu/Zn SOD in hyperoxic lung injury, intravenous Cu/Zn SOD treatment during HTV ventilation was shown to significantly improve lung function, reduce pulmonary oxidative stress and lung inflammation, increase antioxidant defense and NO bioavailability, and help sustain immune homeostasis.

### SOD reduces pulmonary oxidative stress and increases GPx-1 expression in lung

Imbalance of SOD versus GPx-1 often results in the accumulation of hydrogen peroxide and formation of hydroxyl radicals that lead to protein, lipid and even DNA damage [13]. Our data show that intravenous treatment of SOD attenuated HTV ventilation-induced pulmonary hydroxyl radical formation, lipid peroxidation, and significantly enhanced pulmonary antioxidant defense. Though the exact mechanism of SOD-related increasing GPx-1 is unclear, GPx-1 upregulation in response to increased Cu/Zn SOD has previously been observed in a murine cell line study [12]. Reducing oxidative stress and increasing antioxidant activity reduce RI and improve FEV. On the other hand, adding Cu/Zn SOD may not always reduce lipid peroxidation, and that is associated with the level of iron and catalase [54]. H_2_O_2_ derived by Cu/Zn SOD, can cross cellular membrane and form hydroxyl radicals or metal-related reactive species when interacted with redox-active transitional metal ions, such as iron or Cu, via Fenton reaction that leads to lipid peroxidation and cellular injury [17]. Since catalase reduce H_2_O_2_ into H_2_O, increased Cu/Zn SOD in combination with catalase decreases lipid peroxidation.

### SOD attenuates lung inflammation by decreasing MMP-9, VCAM-1 and TNF-α protein expressions and NF-κB inactivation

In this study, HTV ventilation was shown to activate NF-κB and increase VCAM-1, MMP-9 and TNF-α protein expressions, whilst each factor plays a role in lung function impairment, and that was effectively attenuated by intravenous treatment of Cu/Zn SOD.

VCAM-1 overexpression was found in various acute and chronic lung diseases, including acute respiratory distress syndrome [15], ventilator-induced lung injury [8] and pulmonary fibrosis [3]. Cook-Mills et al. showed that VCAM-1 expression in lung is mediated by oxidative stress; antioxidant treatment (vitamins C and E, resveratrol, pyrrolidine dithiocarbamate and *N*-acetylcysteine) can inhibit VCAM-1 signal transduction, thus reducing leukocyte binding to VCAM-1 and decreasing VCAM-1-dependent inflammation [8, 37]. Recently, Segui et al. [71] demonstrated that exogenous Cu/Zn SOD downregulates endothelial VCAM-1 expression that reduces leukocyte rolling and adhesion to vasculatures and leukocyte-endothelial cell interaction.

MMP-9 involves wide-ranging extracellular matrix remodeling, and facilitates inflammatory cell trafficking that contribute to pathological progresses of restrictive lung disease [2] and pulmonary dysfunction in newborn babies [69]. Kim et al. [33] demonstrated that inhibition of MMP-9 attenuates HTV ventilation-induced lung inflammation and alveolar basement membrane damages by restricting neutrophil transmigration and tissue remodeling. Treatment of Cu/Zn SOD has been shown to suppresses MMP-9 expression by interruption of the ROS-NF-κB-dependent pathway [72] and inhibition of the extracellular signal-regulated kinase (ERK) pathway [23].

Upregulation of TNF-α production that depletes cellular antioxidants and increases susceptibility of lung tissues to oxygen radicals [19] is associated with various lung diseases, including asthma, chronic bronchitis, COPD, emphysema [45], pulmonary fibrosis [45], and acute respiratory distress syndrome (ARDS) [55]. Hughes et al. [26] showed that TNF-α can potentiate histamine release at low antigen concentrations in patients with allergic asthma, causing bronchial smooth muscle contraction and increased airway resistance. Also, Wagner [81] showed that TNF-α induces bronchial vasoconstriction, primarily through thromboxane A2 mediated secondary release of endothelin 1. In murine, Choi et al. [7] reported that blockade of TNF-α reduces late-phase airway hyper-responsiveness and airway inflammation, mainly through inactivation of phospholipase A2. In rats, Guery et al. [20] presented that treatment with anti-TNF-α antibody during HTV ventilation significantly reduces lung inflammation, pulmonary capillary permeability and lung edema, and damage to epithelial cells.

A body of evidence suggests that activation of NF-κB plays a crucial role in ventilator-associated lung injury, while inactivation of NF-κB, by means of antibody [5], antioxidant [11], steroid [24] or induced pluripotent stem cells [43], can protect against lung injury. Ko et al. [35] showed that excessive stretch of lung tissue induces non-infectious lung inflammation through activation of NF-κB-interleukin(IL)-6 signaling pathways. Li et al. [39] reported that HTV ventilation caused significant lung injury in wild-type mice, but fails to induce lung injury in protein myeloid differentiation factor 88 (MyD88) deletion mice, in which NF-kB/MyD88 pathway was interrupted. Chiang et al. [5] showed that anti-NF-κB antibody treatment ameliorates lung injury caused by HTV ventilation combined with lung ischemia and reperfusion (I/R) injury or by either insult alone, suggesting the pivotal role of NF-κB pathway in lung injury induced by HTV ventilation and/or lung I/R. In autophagy-deficient mouse strains, where NF-κB was inactivated, Lellouche, et al. [44] demonstrated that HTV ventilation did not induce lung inflammation or lung injury. On the other hand, Lin et al. [41] showed that Cu/Zn SOD attenuates the inflammatory response in human epithelial cells through inactivation of NF-κB and activator protein-1, inhibition of JNK and p38 phosphorylation-mediated VCAM expression, and decreasing TNF-α induced superoxide productions. Also, Yasui et al. [85] reported that exogenously added Cu/Zn SOD regulates neutrophil apoptosis and reduces the caspase-dependent inflammatory response [56], and that is mediated by NF-κB [36].

### Cu/Zn SOD mediates pulmonary vascular resistance and airway smooth muscle relaxation through increasing vascular nitric oxide (NO) bioavailability while reducing iNOS expression

NO plays an essential role in regulating lung function in normal and disease conditions, by which NO modulates pulmonary vascular smooth muscle tone [73] and airway smooth muscle relaxation [65]. NO also involves fibroblast and vascular smooth muscle cell proliferation [21], angiogenesis, and neural development [14]. NO is synthesized in pulmonary endothelial cells from L-arginine by NO synthases (NOSs) existing in three isoforms, namely neuronal (nNOS or NOS-1), inducible (iNOS or NOS-2) and endothelial NOS (eNOS or NOS-3), where eNOS is the primary NOS isoform expressed in the lung [30]. eNOS can be activated by agonists such as acetylcholine [14] and produces NO in a continuous and low-level fashion [73]. As part of the immune defense, iNOS generates NO in large quantities in response to stimuli, including TNF-α, oxidative stress, cytokines and various pro-inflammatory mediators [59]. Evidence indicates that decreasing NO bioavailability plays a major role in pulmonary vasoconstriction and increased airway resistance, resulting from hyperoxia [16], pulmonary hypertension [21] or ARDS [77].

Previous animal and patient studies revealed that large volume mechanical ventilation increases iNOS expression, in addition to ROS production [15, 27, 42]. With the presence of ROS, vascular NO is consumed and NO bioavailability is reduced leading to vasoconstriction [51]. Moreover, NO reacts with ROS to form peroxynitrite, which in turn reacts with lipids, DNA and proteins, causing oxidative damages to cells, resulting in cell necrosis or apoptosis [51]. Previously, Jung et al. [31] showed that vascular NO bioavailability is strongly related to the level of extracellular SOD, since SOD scavenges superoxide anions that compete to bind available NO. In rats, Lynch et al. [46] showed that vascular Cu/Zn SOD deficiency, by means of restricting dietary consumption of copper, can impair endothelial vasodilator function through direct inactivation of NO. Besides, Wu et al. [82] showed that iNOS expression requires NADPH oxidase-dependent redox signaling in microvascular endothelial cells. We showed that intravenous Cu/Zn SOD suppresses iNOS expression in lung, potentially through reducing stimulation by ROS, and steadily increases serum NO concentration during the course of HVT ventilation, supporting increased pulmonary NO bioavailability. We also conducted protein expressions of phosphorylated and unphosphorylated eNOS without finding significant differences among groups.

### Cu/Zn SOD helps sustain pulmonary SP-A and SP-D levels during HTV ventilation

Mechanical ventilation has been demonstrated to affect the secretion and metabolism of pulmonary surfactants [25]. Large tidal volume ventilation, though temporarily increases, eventually suppresses pulmonary surfactant secretion and decreases organized surfactant lipoproteins, and thus alters biophysical properties of surfactants that lead to decrease in lung compliance [52] and impaired pulmonary immune responses [80]. Besides, the presence of pulmonary oxidative stress generated during mechanical ventilation can inactivate pulmonary antiproteases and impair surfactant function [62]. Park et al. [58] showed that 80% reduction in SP-A mRNA expression following ROS stimulation.

In addition to reducing alveolar surface tension, SP-A and SP-D play important roles in pulmonary immune responses. SP-A and SP-D, as pattern-recognition molecules of the collectin family of C-type lectins, mediate pulmonary immune defense through enhancing neutrophil uptake of bacteria [22], and removal of microbes and their debris, pathogens, allergens, dying epithelial cells and phagocytes [80]. Bridges et al. [4] showed that SP-A and SP-D can block low density lipoprotein oxidation and free-radical formation or propagation, so as to serve as potent endogenous inhibitors of lipid peroxidation and oxidative cell damage. Furthermore, Khubchandani and Synder [32] demonstrated that SP-A offsets the inhibitory effects on surfactant function by plasma albumin leaking due to lung injury. Yoshida et al. [89] showed that SP-D reduces alveolar macrophage-associated oxidant production, NF-κB activation, and MMP expression. In mice, Yoshida et al. [89] reported that genetic deficiency of SP-D gene promotes alveolar macrophage infiltration, and increases MMP-2, MMP-9, and MMP-12 expressions and hydrogen peroxide formation in lung, causing progressive pulmonary emphysema. In premature newborn lambs, Sato et al. [67] represented that SP-D administration inhibited ventilation-induced lung inflammation. In this study, HTV ventilation was shown to reduce SP-A and SP-D expression in lung, while intravenous Cu/Zn SOD treatment increased both SP-A and SP-D expressions that were associated with improved lung compliance, decreased MMP-9 and NF-κB expression in lung and improved lung function.

## Conclusions

**Fig. 10:**
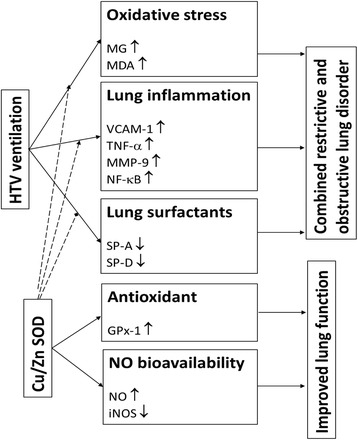
Summary of Cu/Zn SOD on lung function following 5 h of HTV ventilation. (*solid line*: induction; *dashed line*: inhibition)

As summarized in Fig. 10, 5 h of HTV mechanical ventilation induces combined a restrictive and obstructive lung disorder that is associated with increased pulmonary oxidative stress, lung inflammation and inhibited expression of SP-A and SP-D (solid line: induction; dashed line: inhibition). Protective efficacy of intravenous administration of Cu/Zn SOD against HTV ventilation-associated impairment of lung function is related to 4 mechanisms: (1) reductions in pulmonary oxidative stress and improvement in cellular antioxidant defense, as seen by reduced MG and MDA and increased GPx-1 protein expression; (2) decreases lung inflammation, evident by reduced neutrophil and WBC count in the BALF and suppressed MMP-9, VCAM-1, TNF-α and iNOS protein expression and inhibited NF-κB mRNA expression in lung; (3) increased SP-A and SP-D protein expression that improve lung compliance and anti-inflammatory capacity, and (4) increased vascular NO bioavailability that reduces airway resistance and increases air flow rate.

## Abbreviations

BALF: Bronchoalveolar lavage fluid
C_chord_: Chord compliance
ELISA: Enzyme linked immunosorbent assay
FEF: Forced expiratory flow
FEF_25_, FEF_50_, FEF_75_ and FEF_90_: Forced expiratory flow at 25%, 50%, 75% and 90% of FVC
FEV: Forced expiratory volume
FEV_100_, FEV_200_: Forced expiratory volume at 100 and 200 milliseconds, respectively
GPx-1: Glutathione peroxidase-1
HTV + SOD: Intravenous administration during high tidal volume ventilation
HTV: High tidal volume
ICAM-1: Intercellular adhesion molecule-1
iNOS: Inducible nitric oxide synthase
LTV: High tidal volume
MDA: Malondialdehyde
MG: Methylguanidine
MMEF: Maximum mid-expiratory flow
MMP-9: Matrix metalloproteinase-9
NO: Nitric oxide
NOS: Nitric oxide synthase
P_Ao_: Aortic pressure
PEF: Peak expiratory flow
RI: Inspiratory resistance
ROS: Reactive oxygen species
SOD: Superoxide dismutase
SP-A: Surfactant protein A
SP-D: Surfactant protein D
SPs: Surfactant proteins
TLC: Total lung capacity
VC: Vital capacity
WBC: White blood cell
